# Engineering planar antenna using geometry arrangements for wireless communications and satellite applications

**DOI:** 10.1038/s41598-023-46400-9

**Published:** 2023-11-06

**Authors:** Hesham A. El-Hakim, Hesham A. Mohamed

**Affiliations:** 1 Department of Electronics and Communications, Misr University for Science and Technology (MUST), Giza, Egypt; 2 Electronics Research Institute (ERI), Joseph Tito St, Huckstep, El Nozha, Cairo, 11843 Egypt; 3 National Telecommunications Regulatory Authority, Giza, 12577 Egypt

**Keywords:** Electrical and electronic engineering, Engineering, Aerospace engineering

## Abstract

A triple-band microstrip patch antenna designed for the IEEE 802.16e WiMAX, IEEE 802.11a WLAN, C-band downlink communications, and Ku-band radar recent applications is suggested in this article. The planned antenna operates at 2.45, 6, and 14 GHz resonant frequencies. The antenna fulfilled triple-band physical characteristics covering industrial, scientific, and medical (ISM) bands between (2.1–2.8) GHz; (5.6–6.5) GHz for wireless local area network (WLAN) or ultra-wideband (UWB) services; and 12.7–16 GHz for future two-way 5G:6G either broadcasting or mobile satellite communications. To achieve better return loss performance, parametric studies are carried out using Microwave Studio (CST MWS). The proposed antenna is designed on the FR4 as a hosting medium of total size 46 × 38 × 1.6 mm^3^, combined with a planar transmission line (T.L.) feed and defected ground structure (DGS). The simulated antenna’s input reflection coefficient (S11) results and the far-field measurements show good agreement. The fabricated prototype achieves peak gain values of 2.8, 3.8, and 4.7 dBi, respectively, and bidirectional radiation characteristics. A comparative study with other recent publications is implemented to validate the consistency of the design.

## Introduction

Nevertheless, the work on 5G has advanced to 6G and will be properly triggered over the rest of this decade. 5G Advanced will continue to improve end-to-end communication and wireless network performance, bring new efficiencies, and launch the technical foundation for 6G (the next innovation platform for mobile).

In the last few years, wireless communication parts used in 3G, 4G, and 5G networks have undergone astonishing developments. Their applications, like GPS, Wi-Fi, IR, and several others, are developing at enormous speed as well. An essential component of modern wireless communication systems is the antenna, which has displaced cables. Also, the rapid progression of multi-purpose communication devices requires operation in more than one frequency band. Correspondingly, research related to wireless communication systems is increasing, especially in dual, triple, UWB, and multiband antennas^1^. Several demands on the antenna, such as simplicity, size miniaturization, power consumption, and inexpensive cost, are extremely important for cutting-edge wireless technologies. Numerous techniques have been considered to provide a compact antenna size; however, each of them has some limitations. The best compact-size antenna structures that have been considered recently are the micro-strip patch antennas, which can be easily united into wireless communication devices. Generally, the microstrip patch antenna is structured by a thin metal layer attached to a dielectric substrate backed with thin metals and is simply printed on the PCB's surfaces for different wireless terminal equipments^2^. The growth of multi-functional UWB wireless devices uses high data rates, long battery life, and small antenna sizes. These antennas should fulfill the electromagnetic compatibility (EMC) requirements by providing less exposure to electromagnetic (EM) radiation to be safe for human tissues. Moreover, micro-strip antennas play an indispensable part in UWB systems; typically, they aid in the emission of very narrow pulses, each with nearly 1 ns and 1 GHz bandwidth. UWB antennas cover two broad ranges: the lower band, which extends from 3.1 up to 5.1 GHz, and the upper band, which ranges from 5.85 up to 10.6 GHz. The leading wireless technology corporations are now on track with the upcoming wireless systems, such as beyond 5G (B5G)/6G wireless sensor technologies and beyond the 6 GHz frequency (WiFi-6E)^3,4^. GHz Wi-Fi will be frequently engraved into the history of wireless technology by the representatives of the US telecom-regulator Federal Communications Commission (FCC), which voted to utilize 6 GHz in Wi-Fi despite the 1.2 GHz spectrum^5,6^. Wi-Fi 6 diminishes the transmission and delay times of wireless networks queued for users^7^. High efficiency, reasonable gain values, and a small size antenna are preferred for many standards such as IEEE 802.16 (WiMAX), IEEE 802.11ac, and IEEE 802.11n (WLAN) and 5:6 GHz Wi-Fi communication devices^8^. Researchers presented various antenna types to meet the requirements of the most current wireless applications. The well-known microstrip antenna types are target specific, UWB, reconfigurable, dual, triple, and multiband antennas. Moreover, these antenna types have implemented many techniques, like Defected Ground Structure (DGS) and metamaterials, to achieve wideband capabilities^9^. In^10^, a micro-strip antenna containing DGS with a total size of (59.5 × 47 × 1.6) mm^3^ is presented. It achieved triple-band operation at the following resonating frequencies: 1.57, 2.45, and 3.53 GHz. A compact UWB inverted triangular antenna with dual-notch band characteristics is presented in^11^. It offers three operational frequency bands in the UWB range, which are between 3–4.17, 5.33–6.5, and 8.9–12 GHz, respectively. A compact printed UWB antenna with tri-notched characteristics at 3.5, 5.5, and 8.1 GHz with a total size of (30.2 × 25 × 0.762) mm^3^ was introduced in^12^. A quadruple-band notched UWB antenna was presented and fabricated in^13^. It has a size of 32 mm × 30 mm × 1.6 mm and its operation covers four-notched bands at 3.1–3.6, 4.9–6.1, 7.5–8.4, and 10.2–11 GHz, respectively. An UWB MIMO antenna is presented in^14^, composed of a chip antenna, an isolator stub, and a printed circuit board (PCB) with a partial ground plane. A chip antenna patch size of 10 × 10 × 0.8 mm^3^ embedded on FR4 dielectric substrates, besides a PCB of 30 mm × 30 mm, provides operation at 2.45:9 GHz and a maximum peak gain of 4.5 dBi. In^15^, a reconfigurable pattern monopole antenna including two pin diodes with an overall size of 32 × 59 × 0.8 mm^3^ is considered. Its operating frequency band is 3.36 to 3.6 GHz, with realized gain and efficiency of 2.13: 4.93 dBi and 62% to 82%, respectively. In^16^, a patch antenna that operates in UWB-MIMO applications and has (34 mm × 34 mm × 1.6 mm) total dimension with notched triple band characteristics is proposed. It covers the subsequent notched bands (3.3:3.9), (5:6), and (7.4:8.5) GHz with a radiation efficiency lower than 50% and from 75% up to 90% in the other UWB frequency bands. A sub-6 GHz 5G four-element triple-band MIMO antenna is presented in^17^ for wireless applications. It is designed by using FR-4 substrate to operate in the following triple bands: 3.72–3.82, 4.65–4.76, and 6.16–6.46 GHz, with a dimension of 16 mm × 16 mm × 1.6 mm and an average gain equal to 2.5 dBi. In^18^, a four port UWB-MIMO antenna constructed on an FR4 substrate with an overall size of (30 × 30 × 1.6 mm^3^) is presented. The antenna's operating frequency band is (3.1–12) GHz, and it achieved a maximum gain of 6.2 dBi and an efficiency of 87%. A triple-band mm-wave patch antenna is presented in^19^ with triple resonating frequencies at 2.4, 5.5, and 28 GHz. The antenna dimensions are 45 × 40 × 0.508 mm^3^, and it provides gain values of 1.95, 3.76, and 7.35 dBi, respectively. A radiating MPA is introduced in^20^ for conventional sub-6 GHz/5G applications. It uses an Arlon-AD 300C substrate with a 52.92 × 55.56 × 1.2 mm^3^ antenna size and achieves a gain value of 7.15 dBi at a frequency of 5.65 GHz. In^21^, a C-shaped ring antenna integrated with the structure of an artificial magnetic conductor (AMC) and a Styrofoam layer is presented. It has a total size of 54 × 54 × 3.9 mm^3^ and operates at a 2.4 GHz resonating frequency with a 6.21 dB gain and 81% efficiency. As mentioned in^22^, a planner monopole antenna with a partial ground plane is designed with a total dimension of 38 × 50.5 × 1 mm^3^. It achieves operation in the ISM band from 2.075 up to 2.625 GHz with a peak gain of 7.76 dBi and an 80.12% efficiency value. An antenna array module for in-time operation at 3.5 GHz (sub-6 GHz) and 26 GHz mm-wave frequencies is presented in^23^. The antenna modules are fabricated on a commercial FR-4 substrate with a total size of 154 mm × 66 mm × 0.6 mm. It achieves an input impedance matching -10 dB in the sub-6 GHz and mm-wave bands, respectively, to support the feasibility of the recent 5G smartphones.

In this study, a microstrip patch antenna has been designed to enable triple-band operations at 2.5, 6, and 14 GHz. Its double circular ring patches are etched on the upper MPA side, while a partial-rectangular DGS is embedded on the bottom side of the antenna. The whole antenna structure is fabricated using a FR4 (5880) substrate of 46 × 38 × 1.6 mm^3^ dimensions and then combined with a planar T.L. feeder. It is simulated by means of CST MWS, while the antenna prototype structure was developed during the design process. As a result, this antenna was able to be operated in triple-band operation with gain values reaching up to 6 dB, more than 85% radiation efficiency, and bidirectional radiation characteristics.

The substantial features of the planned antenna have been listed as follows: (1) Simple circular radiators and a partial ground plane are loaded on a single square-shaped microstrip patch to achieve triple-band performance. (2) It provides three operational bandwidths using a simple geometrical arrangement, a T.L. feed, and a DGS for ever-changing the operation frequency to lower and upper bands without increasing the antenna size, and (3) the combination of compact size, three operating bandwidths, high efficiency, and high gain recommends future usage of the presented antenna in upcoming wireless communication and LAN network systems. (4) the ${\mathrm{S}}_{11}$ response of the designed antenna is almost modeled by means of series RLC circuits connected in parallel.

The paper is divided as follows: Section "Antenna design and working principle" describes the antenna's design and working principles, along with the analytical design equations. In Section "Simulations results**".**

the simulation results of the planned structures have been offered. Fabrication and tests are then presented in Section "Fabrication and tests"; finally, Sect. 5 presents the conclusion.

### Antenna design and working principle

The presented circular patch micro-strip antenna, along with the rectangular arms and the DGS, are mainly designed to cover the operational requirements at 2.45, 6, and 14 GHz frequencies. The whole antenna structure is embedded on the FR4-5880 commercial substrate; its dielectric parameters are, respectively, 1.6 mm substrate thickness 'h', substrate dielectric constant of 4.4, and 0.02 loss tangent. The planned antenna is constructed of two circular radiator elements supported by four rectangular arms on the upper patch layer, while the DGS is placed on the lower side. As a result, different resonance frequencies have been fulfilled. The designed structure is excited by means of a transmission feed line (T.L.). Since we recognize the importance of mathematical modeling in engineering design, we are dedicated to sharing our antenna design by using mathematical and computer tools to address the design challenges. Mathematical modeling is the act of using equations to represent a real-world situation in mathematical terms and then utilizing those equations to both understand the problem and uncover new features.

In relation to the elementary formulas present in^24^, the design of each circular patch starts by calculating its diameter D by means of the following equations:

1 $$D = 2*T\left[ {1 + \frac{2h}{{\pi T \in_{r} }} \left( {\ln \left( { \frac{\pi T}{{2h}}} \right) + 1.7726} \right)} \right]^{ - 0.5}$$

2 $$T = \left( {8.719*10^{9} )/(\sqrt { \in_{r} } *F_{rc} } \right)$$

where ${F}_{rc}$ is the circular lower cut-off frequency in GHz.

wherein the analysis of the upper rectangular arms and the DGS starts by calculating their widths ${L}_{\mathrm{1,2},\mathrm{3,4},\mathrm{5,6}}$ using the following equation:

3 $$L_{1,2,3,4,5,6} = \frac{C}{{2 F_{r} }}\sqrt {\frac{2}{{ \in_{r} + 1}}}$$

The lengths and then the substrate effective dielectric constant will be calculated by:

4 $$W_{{1,2,3,4,5,6}} = \frac{1}{{2~F_{r} ~~\sqrt { \in _{{eff}} } ~\sqrt { \in _{0} \mu _{0} } ~~~}} - 2\nabla W$$

5 $$\in _{{eff}} = \frac{{ \in _{r} + 1}}{2} + \frac{{ \in _{r} - 1}}{2}~~\left( {1 + 12~\frac{h}{L}} \right)^{{ - 0.5}}$$

wherein, the ${\mathrm{F}}_{\mathrm{r}}$ represents the desired resonant frequency, $\mathrm{L}$ represents the overall antenna width and ϵ_r_ is the substrate relative dielectric constant.

$\nabla \mathrm{W}$ represents the normalized extension of the rectangular patch length which mathematically expressed by the following formula^24,25^:

6 $$\nabla {\text{W}} = 0.412{\text{h* }}\left\{ {\left( {\frac{{ \in_{{{\varvec{eff}}}} + 0.3}}{{ \in_{{{\varvec{eff}}}} - 0.258}}{ }} \right){*}\left( {\frac{{\frac{L}{{\text{h}}} + 0.264}}{{ \in_{{{\varvec{eff}}}} + 0.8}}{ }} \right)} \right\}$$

**Figure 1:**
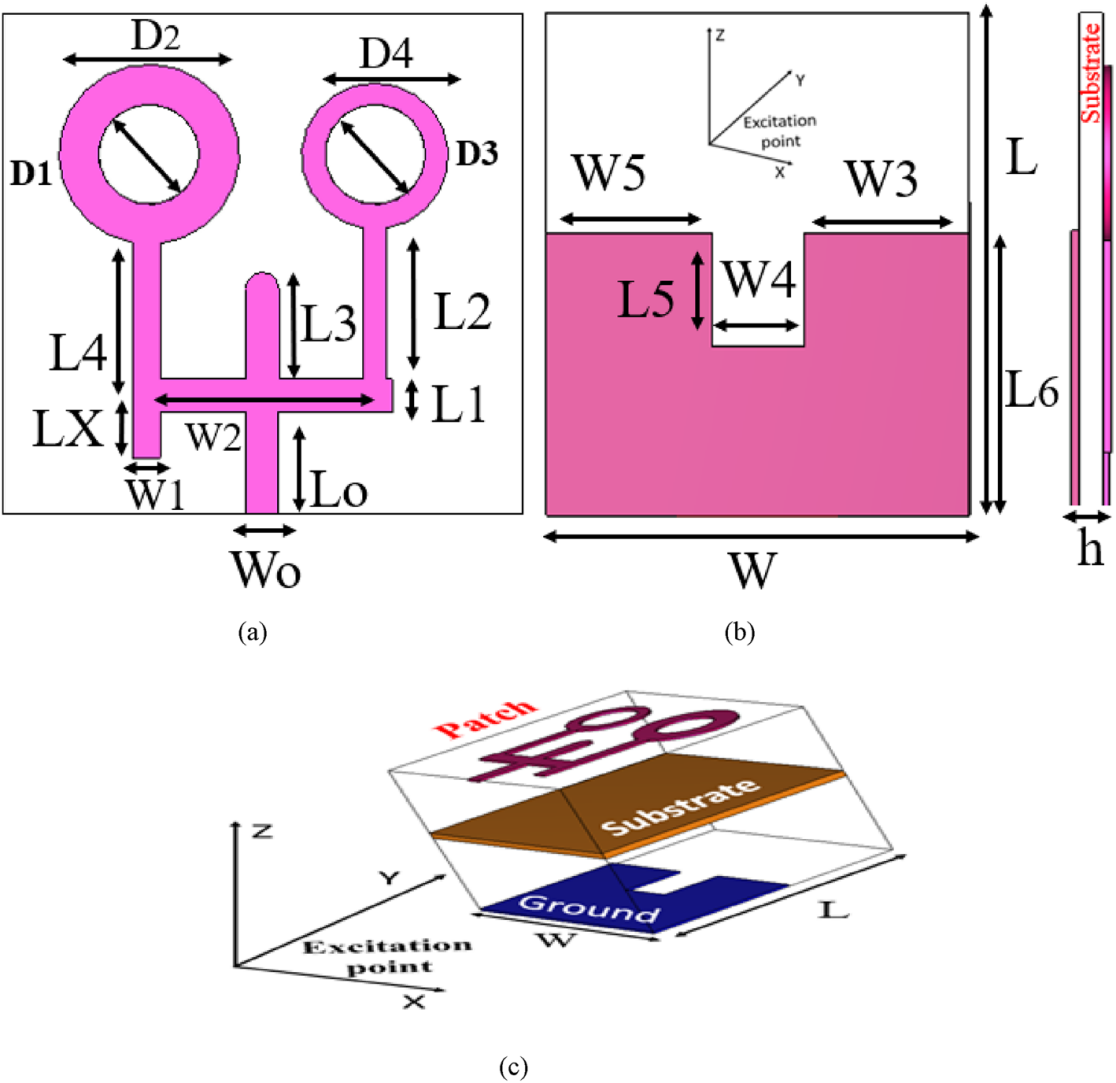
Proposed antenna 2D geometry (**a**) (top view), (**b**) bottom view and (**c**) 3D Geometry.

The initial dimension values for the rectangular and circular patches have been set according to the above equations. Then, the antenna parameters have been parametrically studied through the CST MWS software to get the best values for matching the designed antenna around the required resonances. The overall antenna optimum design parameter values are then listed in Table 1. The designed triple-band microstrip antenna geometry is then illustrated in Fig. 1. The top view exhibits a deep detail of each designed part, starting with the ground plane, up to the coaxial connector nearby the feed line, and ending up at different microstrip patches. Therefore, the top view of the engineered antenna as shown in Fig. 1 has a dimension of 46 × 38 × 1.6 mm^3^. The double circular patches with outer diameters D2 and D4 are taking place on the upper antenna's left and right sides, supported by vertical antenna arms L2 and L4, respectively; each has an inner circular slot. The small slots were loaded on the center of each circular patch of diameters D1 and D3 to improve the performance of the antenna structures and increase the operational impedance bandwidth^25^. To avoid the higher order modes operation and approve the 50Ω microstrip line matching impedance, the metallic T.L. cut-off frequency will be given by^26^.

7 $${\text{ F}}_{{\text{L}}} = \frac{{\text{C}}}{{ \in_{{\text{r}}}^{0.5} \left( {2{\text{L}}_{{\text{O}}} + W_{O} } \right)}}$$

**Table 1:** Proposed antenna dimensions (mm).

Circular patch variables		Rectangular ARMS variables		DGS variables		
Variables	Optimized dimensions	Variables	Optimized dimensions	Variables	Optimized dimensions	
D1	9	W_O_	2.5	W3	18	
D2	15	W1	2.5	W4	10	
D3	9	W2	23.4	W5	18	
D4	13	L_O_	9	L5	10	
L5	10	L1	3	L6	25	
L6	11	L2	13.4	Antenna overall dimensions		
		L3	8	Total length (W)	Total width (L)	Substrate thickness (h)
		Lx	7			
		L4	12.1	40	38	1.6

where c = $3*{10}^{8}$ m/sec represents the speed of light, ϵ_r_ & ${\mathrm{F}}_{\mathrm{L}}$ are the substrate dielectric constant and the feed line frequency, respectively. Also, the estimation of the feed line length was performed by using the next characteristic impedance equation:

8 $${\text{Z}}_{{\text{O}}} = 120{\uppi }/{ }\left\{ {\sqrt { \in_{{{\text{eff}}}} } \left[ {\frac{{{\text{L}}_{{\text{O}}} }}{{\text{h}}} + 1.393 + 0.667{\text{Ln}}\left( {\frac{{{\text{L}}_{{\text{O}}} }}{{\text{h}}} + 1.444{ }} \right)} \right]} \right\}$$

where ${\mathrm{Z}}_{\mathrm{O}}$ is the 50 Ω T.L characteristic impedance and $\frac{{\mathrm{L}}_{\mathrm{O}}}{\mathrm{h}}>1$ ^17^.

The accurate selection of the substrate's width to obtain a 50 Ω microstrip line and keep the minimum loss in microstrip line feed is (${L}_{O}$ = 9) mm^26^. The reflection of the arriving wave is lessened according to the impedance matching methodology, therefore, a microstrip feedline with optimized dimensions Wo and ${L}_{o}$ mm^2^ is loaded on a similar substrate for appropriate excitation. Accordingly, the appointed substrate parameters realize up to 16 GHz operating frequencies without the higher-order modes of operation^26^. The patch impedance ${{\varvec{Z}}}_{{\varvec{L}}}$ is finally designed by using the following Eq. ^24,26^.

9 $${\text{Z}}_{{\text{L}}} = 90{*}\frac{{ \in_{{\varvec{r}}}^{2} }}{{ \in_{{\varvec{r}}} - 1}}{ }\left( {{ }\frac{{\varvec{W}}}{{\varvec{L}}}} \right)^{2} { }$$

As a result, two circular patches with supported arms and DGS are loaded on both sides of the designed microstrip antenna structure with a planar 50 Ω T.L. as shown in Fig. 1.

### Simulations results

**Figure 2:**
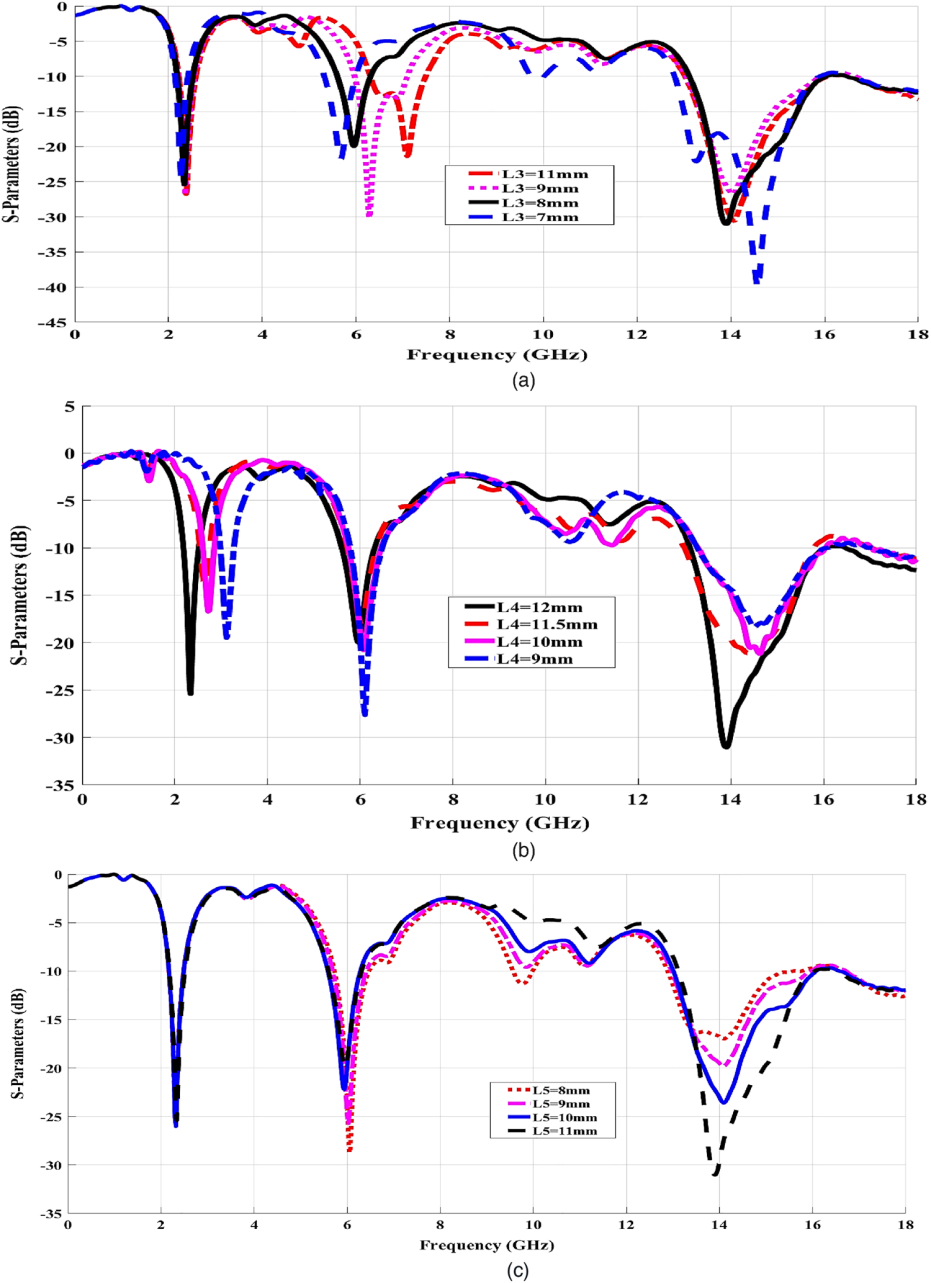

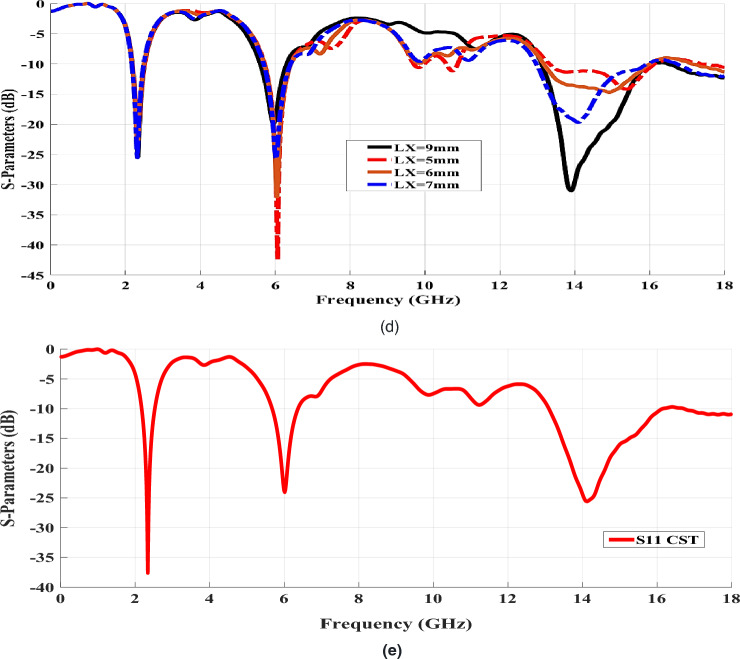
Simulated S11 iterations of the antenna with the different Arm widths. (**a**) L3, (**b**) L4 (**c**) L5 (**d**) LX (**e**) CST Simulated (S11) results of the proposed antenna (0:18 GHz).

Our main objective in the present study is to utilize the performance of both circular and DGS rectangular-shaped patches in the design to achieve the desired resonances. The physical parameter for the circular patches is optimized by means of the parametric study to get their dimensions nearly equal λg/2. While the effect of the DGS rectangular-shaped patch dimensions is producing many features such as increasing the impedance bandwidth, operating frequency shifting, and suppressing the surface wave using the antenna parametric study^27^. It has been observed in CST Microwave Studio through testing the variation in antenna characteristics due to different parameters mentioned^26^ in Table 1. S11 simulation results are then considered through the input reflection coefficient, that relates the antenna absorption measures of the feed power over the total power. Figure 2 shows the effect of changing the widths of antenna arms L3,4,5, and X on the position of the resonance frequencies. The arm lengths were fixed while we constantly varied the arm widths until they resonated at the required frequencies. Figure 2a shows the effect of L3 width values when ranging from 7 mm up to 11 mm. It should be noted that the variation in L3 width tends to change with the location of the second resonance and its bandwidth value. When L3 = 7 mm, the obtained frequency resonance will be at 5.2 GHz. The other resonance will be shifted left or right according to the L3 arm values equal to 8, 9, or 11 mm. Then, the location of the first resonance is changed according to L4 arm values, where L4 can take the following values: 9, 10, 11, 5, and 12 mm. Finally, L5 dominates the location of the third resonance when its values range from 8 up to 11 mm as shown in Fig. 2c. When the width of L5 is increased from 8 to 11 mm, the third notched band is slightly moved from 14.2 to 13.9 GHz. Likewise, the location and the bandwidth of the third resonance are changed, as plotted in Fig. 2d, depending on the variation in LX length. Finally, Fig. 2e reveals the input reflection coefficient S11 of the conventional antenna design. The CST (S11) results of three resonances are represented in Fig. 2e; arrange for perfect choice of upcoming 5G mobile requirements. A higher (S11) power >  − 40 dB has been obtained from the CST simulator at 2.45, resulting from the best matching impedance. Also, 6 and 14 GHz resonances with power greater than − 20 dB imply a matching impedance at these values. It should be noted that the antenna fulfilled the tri-band characteristics covering the ISM bands between 2.1–2.8 GHz; 5.6–6.5 GHz for WLAN or ultra- wideband (UWB) services; and 12.7–16 GHz for future two-way 5G and 6G either broadcasting or mobile satellite communications^1–5^. The circularly loaded radiators besides the rectangular DGS are then providing operation in triple-frequency resonances, as plotted in Fig. 2e.

#### Surface current distribution on the proposed antenna structure

**Figure 3:**
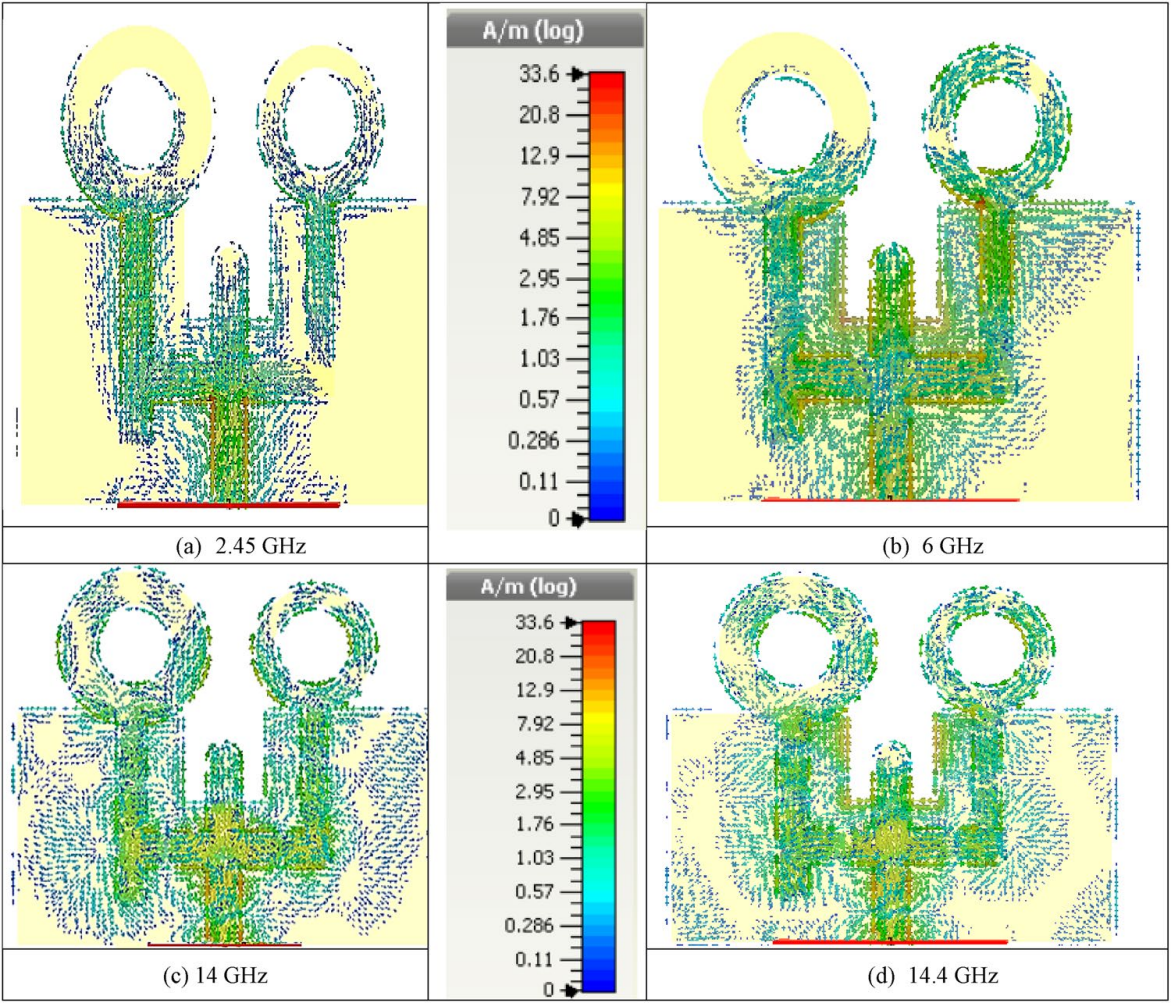
Surface current distribution at different frequencies for the proposed design.

For further description of the designed antenna's performance, the surface current distribution of the designed antenna was outlined at various frequencies by means of a CST simulator. Thus, the surface current distributions at circular patch-loaded.

radiators at some designated frequencies are illustrated in Fig. 3 where the red and blue colors represent the strongest and lowest current distribution areas, respectively.

 As the main mechanism of the electromagnetic radiation caused by the current distribution around the surface of antenna feedline and within the edges of the circular patches. In Fig. 3a, at a frequency of 2.45 GHz, the surface current was mainly distributed around the edges of the antenna feed line and with a lower degree on the inner of the upper circular patches. It should be noted that the surface current is distributed at the edges of the two radiating elements (circular patches) and around the feed line at 6 GHz, as in Fig. 3b. Moreover, the crucial influence of the constructive coupling on different antenna patches is also depicted in Fig. 3b. As a result of this resilient coupling, much energy is attached to the different micro-strip line elements, and the surface currents distribution is then at 6 GHz. The surface current is mainly distributed on the edge of the feed line at 14 GHz, as plotted in Fig. 3c. Finally, the feed line and the edges of the DGS middle inner slot are responsible for the distribution of the surface current at 14.4 GHz, as shown in Fig. 3d.

Consequently, the intensive surface currents distributed on the edges of the inner slot (inserted at the DGS rectangular patch) make them more resourceful than the feed line. Nevertheless, the shift around the frequency’s resonances takes place due to the effect of DGS underneath the various patches; these added resonances achieve a wider triple-band performance based on optimizing the DGS to increase the operating bandwidth around the resonating frequencies^28^.

### Fabrication and tests

**Figure 4:**
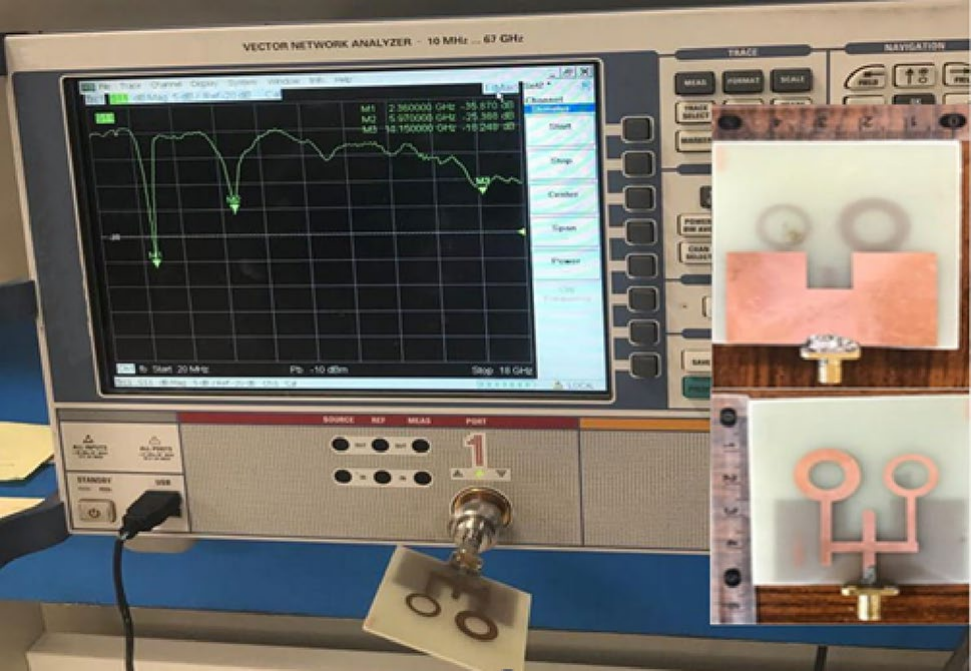
Measuring manufactured antenna input reflection coefficient (S11) values by a VNA.

**Figure 5:**
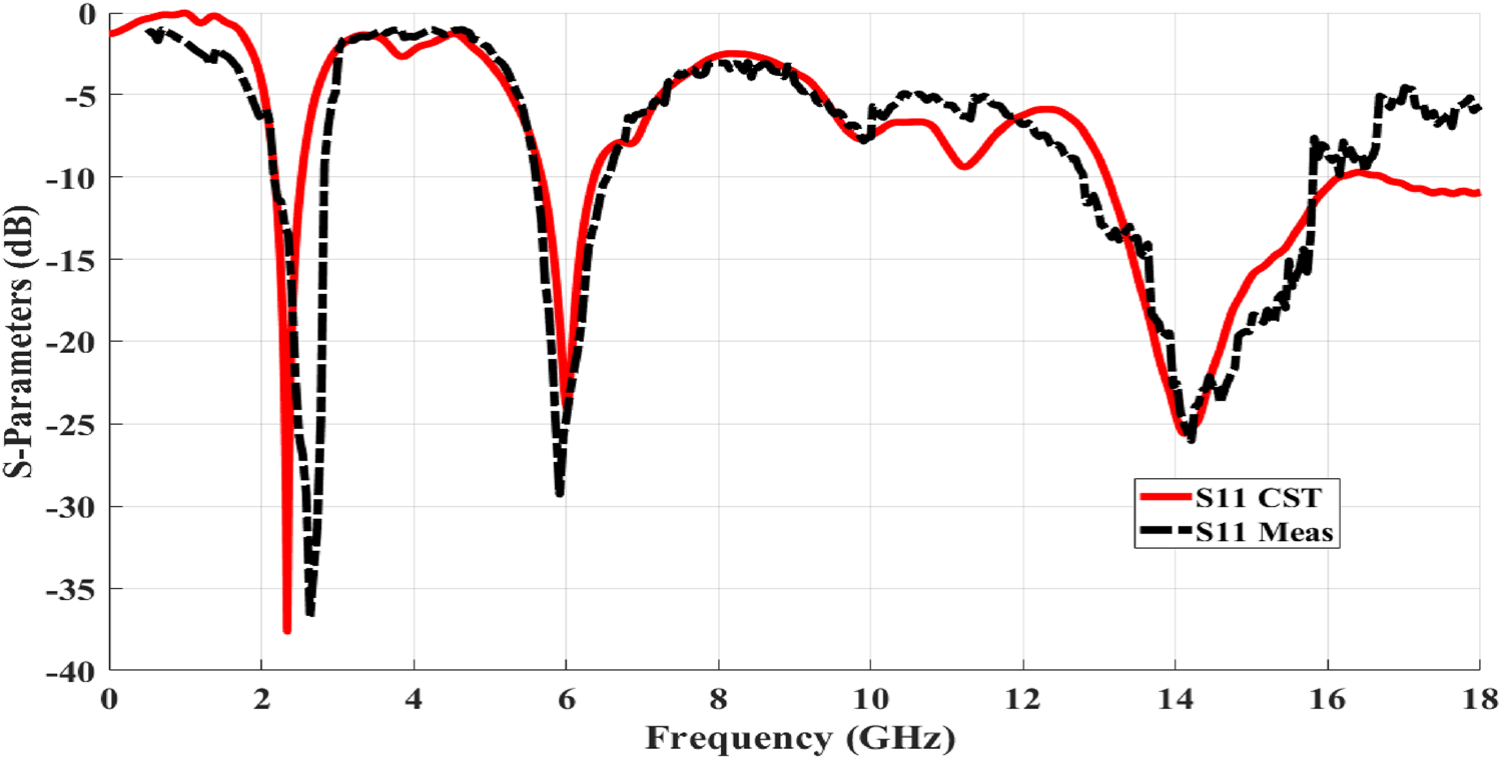
Measuring versus simulated antenna input reflection coefficient (S11) values.

As a result of the inherent analysis, good-looking surface current distribution features, and the various resonances, a straightforward perspective of the proposed antenna's performance is realized. The manufacturing process was then executed to confirm the preceding simulation results and validate the physical characteristics of the proposed design. The input reflection coefficient (S11) parameters of the fabricated antenna prototype were measured using the 50 Ω port of the ZVA 67 Rohde and Schwarz vector network analyzer (VNA). The test arrangement is depicted in Fig. 4 where the antenna’s terminal is directly connected through a coaxial cable to (VNA) port 1 as listed in^29^. Figure 5 represents the measuring antenna (S11) versus frequency over the operating bandwidths. The comparison between the simulated and measured (S11) results is evaluated through the contrasts between them. After the fabrication and measurement of the antenna, the (S11) physical characteristics at the assigned frequencies were clearly achieved. Practically, the frequency bands are cantered at 2.45, 5.9 and 14.4 GHz. Regarding the comparison between the computed results and the measured ones, as shown in Fig. 5, the results point to good consistency between the CST simulation and the measurement results within the overall frequency ranges. Also, wider impedance bandwidths over the assigned resonances are realized by using the FR-4 substrate due to the existence of DGS^25^.

**Figure 6:**
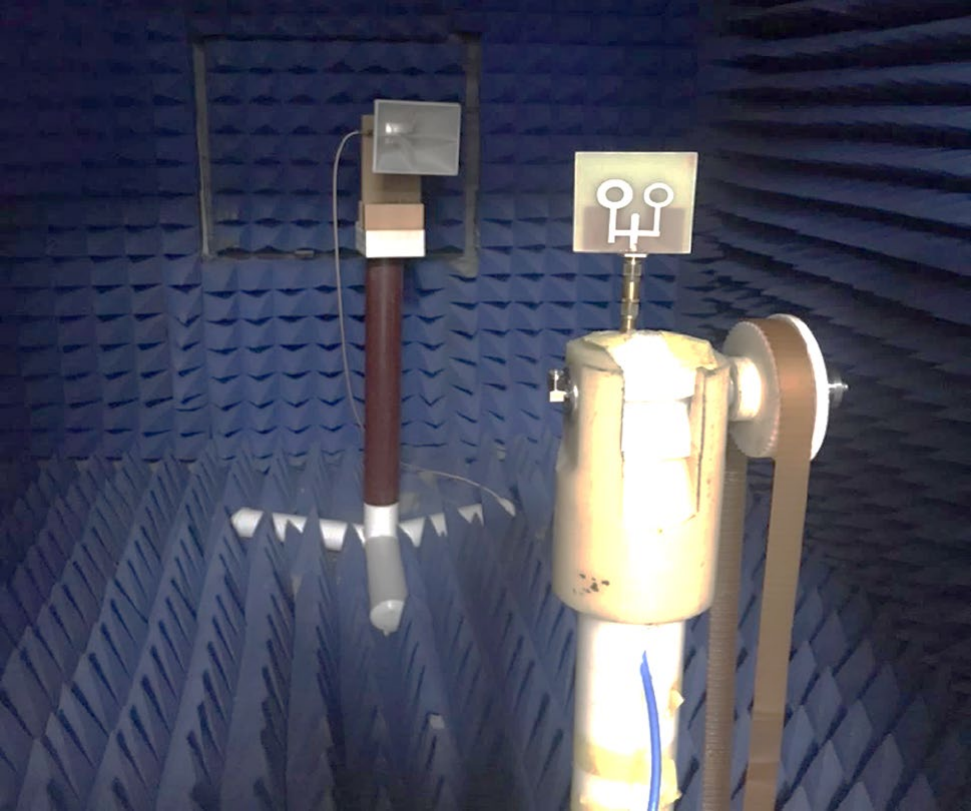
Anechoic chamber measurement setup for the fabricated prototype.

Further inspection was done on the proposed antenna by examining the 2-D radiation characteristics at some valid frequencies and antenna gain values over the related bandwidths. The anechoic chamber is utilized during the measurement as depicted in Fig. 6. The double-ridged horn antenna operates until 20 GHz is utilized as the transmitting end, while the fabricated prototype is fixed at the receiving terminal with 2 m of space between them to achieve the far-field requests. The line-of-sight direction is realized between the transmitted and received ends. The antenna prototype is then tested by rotating it in both the horizontal and elevation planes. By connecting the fabricated antenna to port 1 and horn antenna (reference)to port 2 of the (10 MHz to 67 GHz) Network Analyzer, the gain is taken out by measuring S21^30^. Table 2 demonstrates the tested and normalized simulated radiation patterns at four different frequencies in the E (Phi = 0°) and H (Phi = 90°) planes.

**Table 2:**
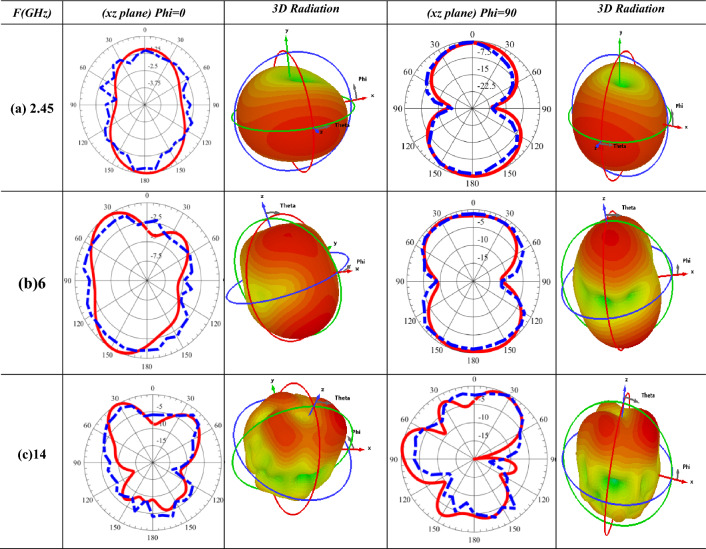
Measured and simulated radiation patterns 2-D radiation characteristics at E and H planes for xz plane and yz plane of the designed antenna: measured (______ Solid line)) and simulated (––- Dashed line) for three different frequencies (a) 2.45, (b) 6, and (c) 14 GHz.

The radiation patterns at 2.45 GHz (first band), as depicted in Table 2a, have practically bidirectional pattern in the E plane and a bidirectional pattern in the H plane. While the proposed antenna has an almost bidirectional pattern in the E-plane and a bidirectional pattern in the H-plane at 6 GHz (second band), as tabulated in Table 2b. Lastly, at 14.4 GHz (third band), shown in Table 2c, the antenna shows a close to bidirectional pattern in both E-plane and H-plane. Accordingly, a broadside direction was achieved in both planes, either in simulated or measured 2-D radiation patterns at each band, as represented^19^ in Table 2.

**Figure 7:**
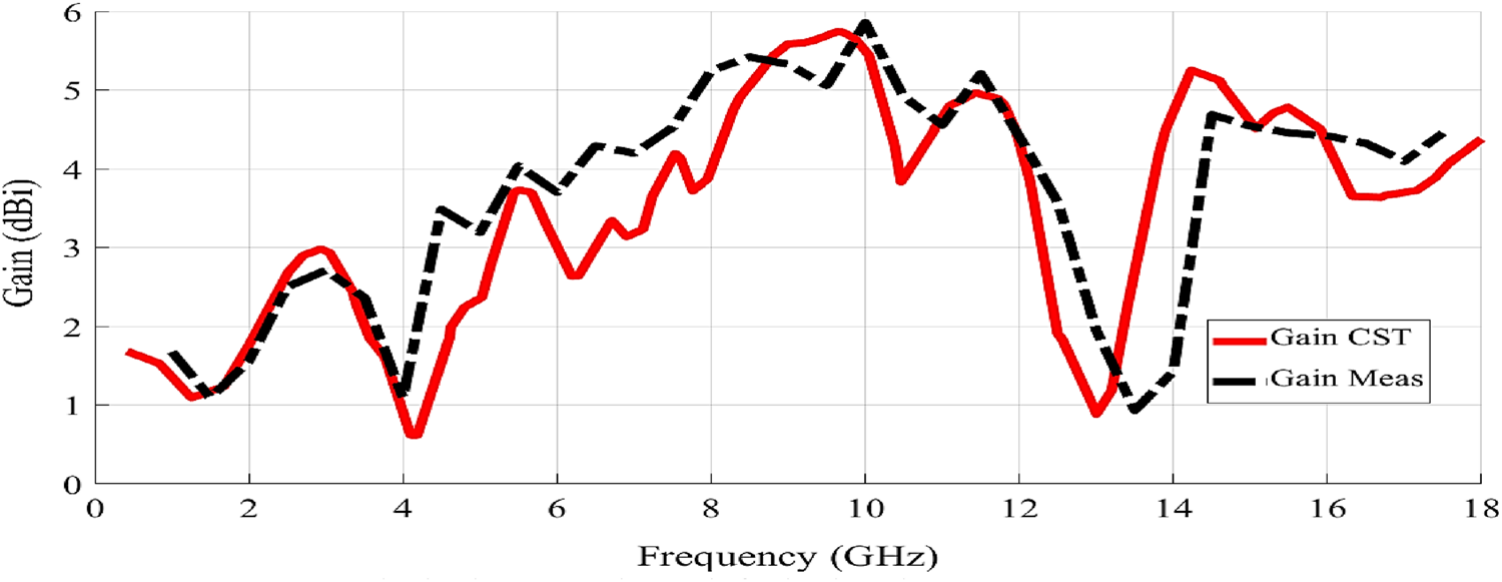
Simulated vs measured 2-D gain for the planned antenna 0:18 GHz.

Additionally, the simulated versus measured 2-D gain values of the fabricated antenna were verified in Fig. 7, where a maximum gain of almost 5 dBi is achieved at 14.4 GHz, as shown in Fig. 7. It was noted that the fabricated prototype preserves the same characteristic performance with gain values ranging from 3 up to 5 dBi at the operation frequencies of 2.45, 6, and 14.4 GHz, respectively^29^. As shown Fig. 7, the fabricated antenna configuration has a wide range of variation in the gain magnitude in the designated frequency bandwidths. These substantial variations are due to different antenna elements causing unlike edge impedances. Hence, the highest gain is achieved with better impedance matching, while the lowest gain is due to lower impedance matching^31^.

**Figure 8:**
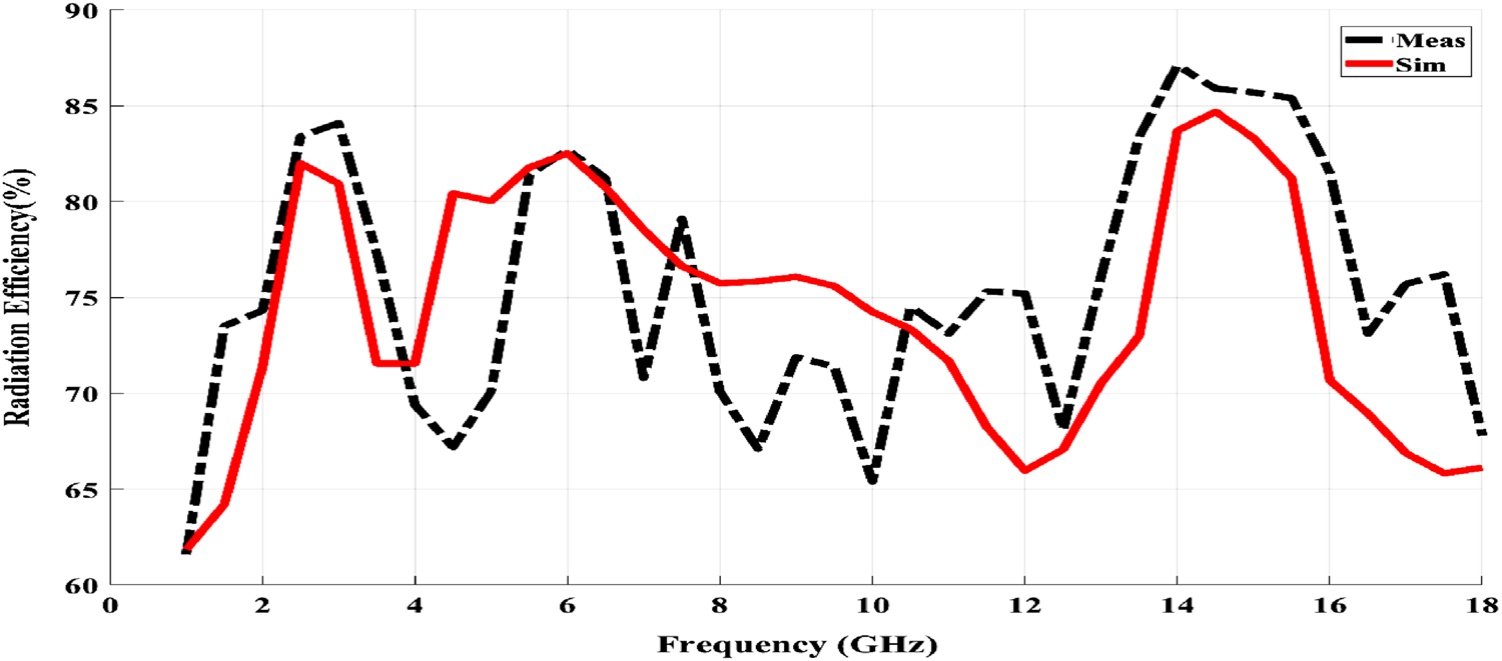
Simulated and measured radiation efficiency for the proposed antenna 0:18 GHz.

The measured and simulated antenna radiation efficiency over the overall frequency range is illustrated in Fig. 8. In the triple frequency bands, the simulated antenna radiation efficiency is between 80 and 85% for the three bands. Moreover, the measured radiation efficiency values are around the simulated ones at 2.45, 6 GHz, and they slightly increased to 87% at 14 GHz.

### Equivalent circuit model for the proposed design

**Figure 9:**
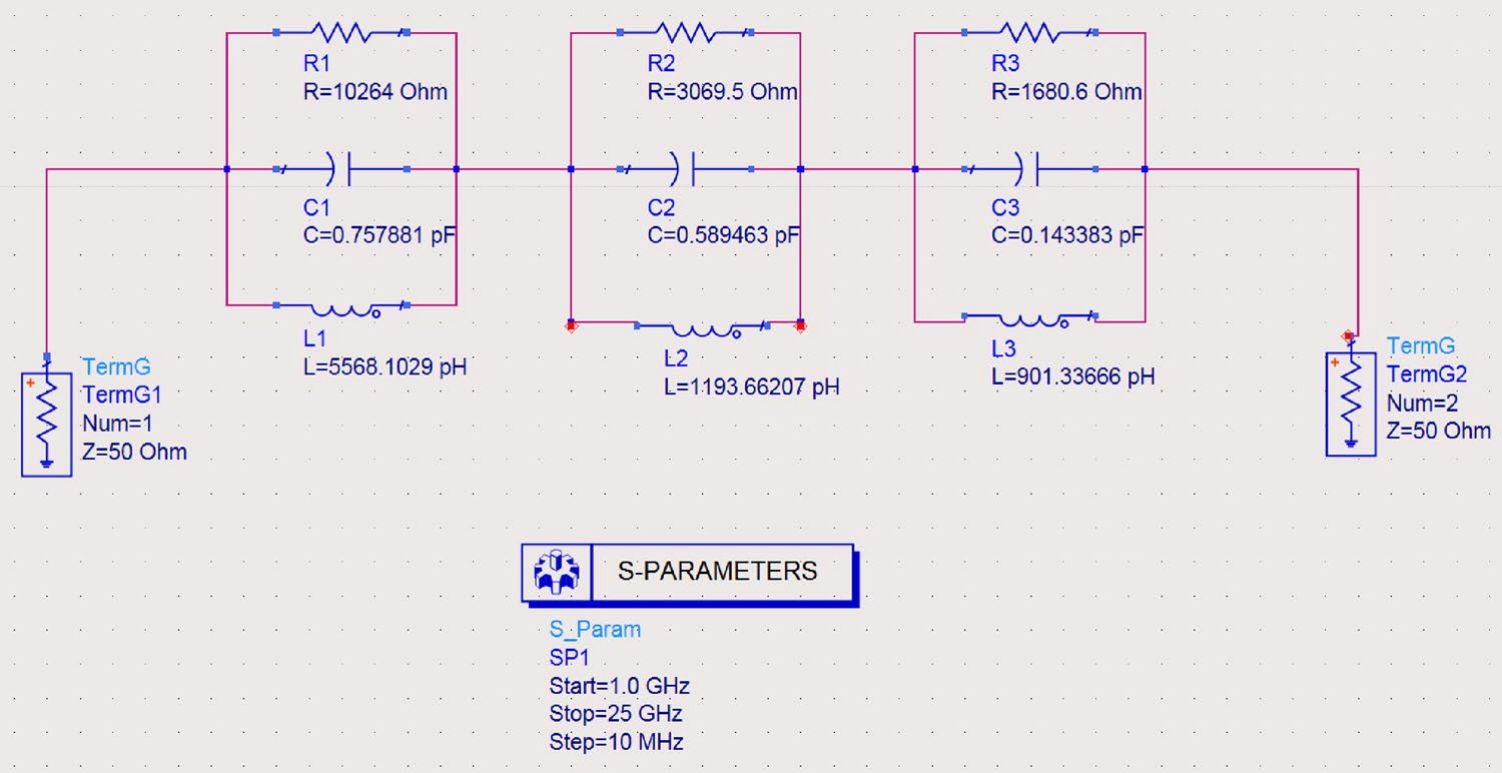
Equivalent circuit model of the proposed micro-strip antenna.

**Figure 10:**
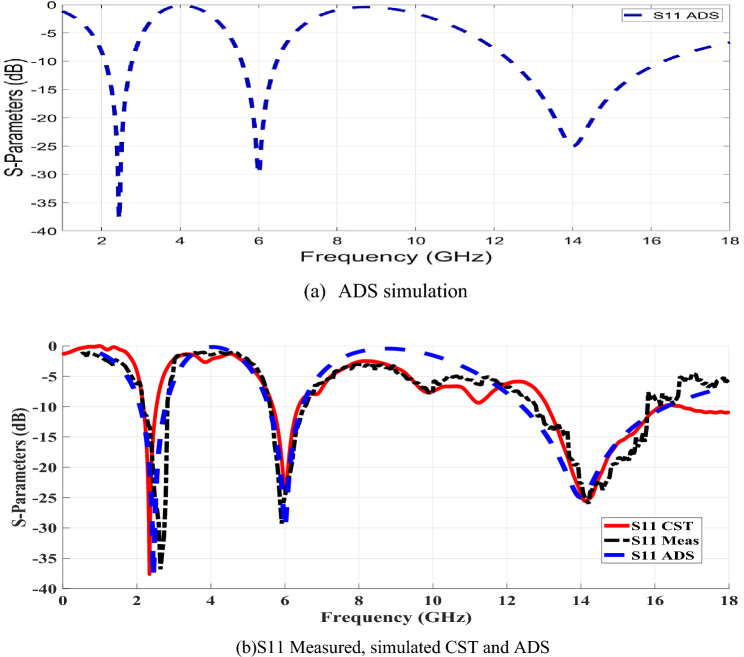
Measuring VS Simulated antenna input reflection coefficient S11 values.

Generally, the equivalent circuit model can be done using a parallel or series RLC circuit, where the microstrip patch antenna performs a bandpass filter. The antenna attenuates all frequencies except those in the desired frequency range. As a result, the proposed antenna's input reflection coefficient (S11) has been equivalently modeled using parallel resonant circuits via the Advanced System (ADS) toolbox. The equations relating the required bandwidth and resonant frequency with R, L, and C components are completely described in^32,33^. Figure 9 depicts RLC circuits, which are made up of R, L, and C connected in parallel. In Fig. 9, the circuit modeling of the operating bandwidth is presented, where three parallel circuits with different values of L, R, and C are used. These arrangements are equivalent to connecting three band-pass filters in a parallel network, each with various R, L, and C values. Furthermore, the values of each R, L, and C network section related to its operating bandwidth are mentioned in Table 3. Figure 10 depicts the (S11) response of the three parallel RLC networks simulated by ADS and fabricated antennas at the 0:18 GHz frequency range. The S11 results over the whole operating bandwidth have been shown in Fig. 10a, which are equivalent to the R, L, and C parameters carried by the ADS toolbox. Besides, the simulated CST and measured (S11) results are also plotted in Fig. 10b. Generally, these results represent the configuration of three parallel RLC networks at 2.45, 6.6, and 14 GHz resonance frequencies. It should be noted that the calculations performed with a series RLC resonant circuit are strongly matched with the corresponding simulated and measured results obtained by means of the CST tools and VNA, respectively.

**Table 3:** RLC circuit values for the fabricated microstrip antenna.

Resistors (ohm)	Capacitance (PC)	Inductance (PH)
R1 = 10,264	C1 = 0.757881	L1 = 5568.1029
R2 = 3069.5	C2 = 0.589463	L2 = 1193.66207
R3 = 1680.6	C3 = 0.14383	L3 = 901.33666

In conclusion, Table 4 illustrates a comparison between the planned design and some recently published results. As depicted in Table 4, the fabricated microstrip antenna attained better (S11), gain, efficiency values and radiation characteristics than the formerly designed structures. The proposed antenna exhibited excellent performance within the operational frequency range between (2.1–2.8), (5.6–6.5), and (12.7–16) GHz. Thus, this manufactured prototype can be used for cutting-edge 5G/6G microstrip antenna technologies.

**Table 4:** A comparison between the proposed antenna and other published papers.

Ref	Size (mm^3^)	Size (λ_g_)^2^	Substrate-type	Resonance freq/BW (GHz)	No. of operational-bands	Radiation pattern characteristics (S11)/Gain (dBi)	Application
^10^	59.5 × 47 × 1.6	1.74 × 0.98	RT ∈ _r_ = 2.2	1.57/0.02 2.45/0.06 3.53/0.06	3	− 20/4.6 dBi on average	GPS, ISM and WiMAX
^11^	62 × 64 × 1.6	1.26 × 0.86	FR4 ∈ _r_ = 4.4	3.5/1.2 6.1/1.17 9.25/3.1	3	− 28/4.75 dBi on average	UWB
^19^	45 × 40 × 0.508	2.1 × 1.93	RT ∈ _r_ = 2.2	2.4/0.38 5.5/1.29 28/3.16	3	− 18/5 dBi on average	4G/5G
^30^	40 × 30 × 1.6	1.7 × 1.45	FR4 ∈ _r_ = 4.4	9.65/0.177 11.68/0.906 16.54/1.966 21.28/2.9 29.7/5.095	Multi-band	− 25/4 dBi on average	Satellite Communications
^34^	52 × 52 × 1.52	1.8 × 0.98	RT3003 ∈ _r_ = 3	3.4/ ~ 0.1 3.9/ ~ 0.2	2	− 28/4.5 dBi on average	sub-6 GHz n77 band
^35^	57.3 × 75.85 × 1.6	1.8 × 2.74	FR4 ∈ _r_ = 4.4	2.4/0.058	1	− 27/4.3 dBi on average	Wi-Fi, Bluetooth, and ZigBee
^36^	83 × 56 × 1.56	2.29 × 2.94	FR4 ∈ _r_ = 4.4	2.46/0.38 3.51/0.19 5.55/0.3	3	− 22/3.86 dBi on average	Wi-Fi/WiMAX and WLAN
^37^	3 antennas Each with 28 × 37 × 1.6	1.64 × 1.25	FR4 ∈ _r_ = 4.4	3.47/1.35 5.5/1 9.45/3.5	3	≥ − 10 dB/3.8dBi on average	cognitive radio communication in 5G WLAN,LTE
This work	40 × 38 × 1.6	1.3 × 1.02	FR4 ∈ _r_ = 4.4	2.46/0.38 3.51/0.19 5.55/0.3	3	− 29/5 dBi on average	GPS, ISM ,WiMAX, 5G/ sub-6 GHz and satellite communications

## Conclusion

In this work, an UWB microstrip patch antenna operating in triple-band, covering 2.45, 6, and 14 GHz resonance frequencies was engineered. These frequencies have implications for modern wireless technologies, especially 5G/6G applications. The design steps of the proposed antenna are discussed in detail. It is based on reducing the overall antenna size by using circular-radiator patches besides DGS, all printed on the same substrate. The strong couplings between both driven and parasitic patches offer triple-band operation with three different frequency resonances. Moreover, this configuration experimentally achieves proper impedance matching, gain, and efficiency values. The radiation characteristic enhancements of the proposed antenna in the overall operating frequency ranges are thus fulfilled. The fabricated prototype exhibits triple-band antenna performance at the definite frequencies of input reflection coefficients more than − 10 dB. The results show that the efficiency and gain values of the planned antenna reach up to 87% and almost 6 dBi with good bi-directional radiation characteristics.

## Data Availability

All data generated or analyzed during this study are included in this published article.
